# Surveillance of nosocomial infections in the Yaounde University Teaching Hospital, Cameroon

**DOI:** 10.1186/s13104-016-2310-1

**Published:** 2016-12-08

**Authors:** Julienne Stéphanie Nouetchognou, Jérôme Ateudjieu, Bonaventure Jemea, Edmond Nzene Mesumbe, Dora Mbanya

**Affiliations:** 1 Denis & Lenora Foretia Foundation, P.O. Box 14315, Yaounde, Cameroon; 2 M.A. SANTE (Meilleur Accès aux Soins de Santé/Better Access to Health Care), P.O. Box 33490, Yaounde, Cameroon; 3 Department of Biomedical Sciences, University of Dschang, P.O. Box 67, Dschang, Cameroon; 4 Division of Health Operations Research, Ministry of Public Health, Yaounde, Cameroon; 5 University Teaching Hospital of Yaoundé, Yaoundé, Cameroon; 6 Faculty of Medicine & Biomedical Sciences, University of Yaoundé I, Yaounde, Cameroon

**Keywords:** Nosocomial infections, Patient safety, Epidemiology, Surveillance, Cameroon

## Abstract

**Background:**

Nosocomial infections (NI) represent a real public health problem in developing countries. Their surveillance is recommended to provide needed information for better control. The aim of this study was to describe the frequency and distribution of NI in the Yaoundé University Teaching Hospital (YUTH).

**Methods:**

It was a longitudinal and descriptive study targeting hospitalized patients in the intensive care, gynaecological, surgical and neonatal units. Each consenting patient was administered a questionnaire at the beginning of the study and followed up daily for the duration of their hospitalization using a standardized grid to detect all nosocomial infections. Cumulative incidence was used to estimate NI frequency.

**Results:**

There were 307 patients included. The cumulative incidence and specific mortality rate of NI were 19.21% (16.9–21.5) and 28% (16.2–42.5) respectively. Septicaemia (20.34%), infection of the skin and soft tissues (20.34%) and urinary tract infections (15.25%) were the most frequent type of NI. *Klebsiella* spp. was the most frequently isolated bacterium (27%).

**Conclusion:**

Nosocomial infections contribute to high hospital morbidity in the Yaounde University Teaching Hospital. Strategies need to be identified for a sustainable and continuous monitoring of NI in all health facilities of Cameroon. In addition, Further studies should identify NI determinants and interventions for efficient and better control.

## Background

Healthcare-associated infections (HAIs) or nosocomial infections are infection contracted by a patient at least 48 h after his admission; they can be recognize on clinical, biological, microbiological and radiological plans [1]. They are a common causes of morbidity and mortality in developed and developing countries [1–4]. A minimum of 190 million people are hospitalized each year worldwide and 9 million of them acquire at least one episode of HAIs [1, 5]. The prevalence of nosocomial infections depend on the level of development of the health system. Thus, in France and USA the prevalence of NI is estimated to vary between 6 and 7% and between 3 and 5% respectively [5, 6]. In Africa this prevalence reaches 25% of hospitalized patients [7, 8]. It is even expected to be worse since no data are available for countries that are expected to have the highest burden of the problem. In Cameroon, few investigations have been conducted but an isolated study done in 2010 stated this prevalence at 20.74% [9]. To reduce the burden of the problem; WHO recommended that HAIs surveillance and prevention should be systematically integrated in all health system [1, 4]. This recommendation has been implemented by many countries worldwide, including USA, France and some developing countries, thus promoting surveillance and prevention of HAI in their hospitals. This has allowed for the identification of NI risk factors, and effective interventions that helped in reducing their incidence by nearly 30% in some countries and 55% in Africa [1, 10].

Cameroon has not yet adopted a national policy on surveillance, prevention and management of nosocomial infections. And no data on the incidence of cases is asked from health facilities during the routine national surveillance. The situation in each facility depends on the knowledge of health authorities on the issue, their motivation and the availability of resources to integrate this activity into their action plan. To attract the attention of policy makers on the magnitude of the problem and possible solutions, this study was carried out to determine the incidence of NI in one of the referral hospitals in Cameroon.

## Methods

### Study design

This was a descriptive and longitudinal study targeting hospitalized patients of intensive care, gynaecological, surgical and neonatal units. Each consenting hospitalized patient or guardian (for neonates) was administered a questionnaire at the entry and followed up using a preconceived grid for the duration of his hospitalization, seeking for any symptoms or signs of nosocomial infections. Cases were provided with routine care by the health personnel including sample collection and testing for etiological investigation, and where applicable conducting tests to isolate bacteria and administering indicated therapy. The surveillance was conducted from 1st September 2013 to 09th March 2014.

### Study site

The Yaoundé University Teaching Hospital (YUTH) has a capacity of 200 beds for hospitalization with a patient’s load of 500–1000 per week. This institution has not only the charge of taking care of many patients who require a long period of hospitalization, but also the charge of training physicians to serve the health care system of the country.

At the time of the study it had an Emergency unit, a Surgical ward and its sub-specialties as well as three operating theatres, Obstetrics–Gynaecology, Paediatrics, Internal Medicine, intensive care units, Laboratory, the outpatient Department, the Mortuary, the Pharmacy, and the Hygiene and Maintenance Unit. It counted 325 health staff consisting of 62 physicians, 236 nurses and 27 laboratory technicians. For the year 2013, the YUTH recorded 6187 in-patients for a total of 25,869 days. The average stay was thus 4.18 days and the average occupancy rate of beds was 39%.

### Sampling

We selected the four services that have been documented in the literature as services with higher risk of developing a NI: gynaecological, neonatal, surgical and intensive care units. In each service, all hospitalized patient were eligible for the study, but only consenting ones, hospitalized for at least 48 h were included.

### Study procedures

Data collection tools were conceived by the study team including 1 Master student in Epidemiology and Public health, and 3 Medical Doctors (1 specialist in haematology, 1 Epidemiologist, and 1 specialist in internal medicine), pretested in the Dschang district hospital, reviewed and validated.

Permission to carry out the project was obtained from the director of YUTH and heads of the selected units, prior to patients’ enrollment. Informed consent was obtained from all patients hospitalized in the targeted services (Hospitalisation was defined as spending at least 48 h in the service) before being included into the study. Each included patient was followed up during hospitalization until their discharge from the hospital. A questionnaire was administered at the entry to the patient or his guardian and a grid was used for daily monitoring. Data were collected on socio-demographic characteristics of the patients, their medical history, invasive devices for their care, and the occurrence of NI. Medical history of patients included in the surveillance was assessed by screening of their medical record as shown in the Table 1 below. The HIV status was defined using the results of the HIV testing early done by the patient or done at the time of the admission in the hospital.

**Table 1:** Data collected on medical history

Criteria	Measure/test	Abnormal cut-off used
Anemia	Hemoglobin	<13 g/dl in male; Hb <12 g/dl in female; Hb <10 g/dl in pregnant women and Hb <15 g/dl in neonates
Diabetes	Blood sugar at 24 h intervals	<1.26 g/l or the patient known as diabetic
Hypertension	Blood pressure	>140/90 mmHg or a patient known to be hypertensive
Obesity	Body mass index	>25 kg/m^2^
Premature births	Gestational age and birth weight	Gestational age <37 weeks and low birth weight as birth weight <2500 g

The recruitment period was 5 months from the 1st September 2013 to 08th February 2014, enabling the last participant to be followed up for at least 30 days.

Clinical diagnostic of NI was based on criteria listed in the Table 2.

**Table 2:** Criteria used in the diagnostic and definition of nosocomial infections

Type of infection	Clinical/radiological criteria	Minimum criteria for the diagnosis
Surgical site infection	Pus (1), abscess or cellulitis (2), sero-bloody flow (3), redness and/or heat (4), Fever (temperature ≥38 °C) (5)	(1) or (2) or (3) or the diagnosis evoked by the clinician
Urinary infection	Lumbar or sus-pubic pain (5), dysuria or pollakiuria (6), fever (temperature ≥38 °C) or chills (7)	(5) or (6) + (7) or isolate fever of the newborn or the diagnosis evoked by the clinician
Pulmonary infection	Fever (8), cough (9), purulent expectoration or secretion (10), pulmonary auscultation signs (11), clinical sings of pleural effusion (12), radiological sign of pneumopathy or abscess (13)	(11) + 3 other criteria, or (12) + 3 other criteria or two criteria after endotracheal maneuver or signs of respiratory distress in newborns , or the diagnosis evoked by the clinician
Infection of the catheter	Pus (14), sero-bloody flow (15), redness and/or heat (16), fever ≥38 °C dropping after removal of the catheter (17)	At least one of the criteria or the diagnosis evoked by the clinician
Septicemia	Fever or chills (18)	(18) or the diagnosis evoked by the clinician

### Data analyses

The diagnostic of nosocomial infection in the study was based on clinical symptoms. The overall frequency of nosocomial infection was estimated by calculating the cumulative incidence (number of cases of NI/Total number of patients included) and its distribution per service, and the incidence rate (number of NI case during the hospitalisation period/sum of the duration of observation for each patients). The specific mortality rate was also estimated, as well as the proportion of the different bacteria isolated from the participants.

### Laboratory procedures

Microbiological specimens were processed according to good laboratory practice and standard methods for identification at the bacteriological laboratory of the University Teaching hospital center of Yaounde. The antimicrobial susceptibility test was determined by the Kirby-Bauer disk diffusion method following the National Committee of Clinical Laboratory Standards (NCCLS) for agar diffusion tests [11]. Test results were only validated in the cases where inhibition zone diameters of the control strains were within performance ranges. Data were analyzed and resistance included combined intermediary and resistance results.

### Ethics and consent

The study was examined and approved by the Cameroon National Ethical Review Committee (No 2013/11/376/L/CNERSH/SP).Written informed consent was sought from each participant prior to inclusion in the study.

### Consent for publication

Upon accessing consent to participate, consent on publication of results from the study was also obtained from the participants.

### Availability of data and materials

The dataset of this study will not be shared because the Ethical guidelines prohibit researchers from providing their research data to other third-party individuals. The data used for the study are not openly available.

## Results

### Characteristics of the study population

A total of 307 patients were included in the study among which 138 (44.95%) were males and 169 (55.05%) females. The average age was of 40.28 ± 1.13 years.

Regarding the medical history of the participants, 24 (7.9%) had HIV infection, 34 (11.2%) were known to have hypertension. Table 3 describes predisposing factors and exposure of participants to possible sources of NI transmission.

**Table 3:** Description of the study population: frequencies of intrinsic and extrinsic factors recorded

Characteristics	Neonatology		ICU		Gynaecology		Surgery		Total	
	Number (total = 54)	%	Number (total = 51)	%	Number (total = 71)	%	Number (total = 131)	%	Number (total = 307)	%
Intrinsic factors										
Anaemia	NA*	NA*	6	12	0	NA	1	0.8	*7*	*2.3*
Diabetes	NA*	NA*	8	16	1	1.4	7	5.5	*16*	*5.3*
Hypertension	NA*	NA*	23	46	2	2.9	9	7.0	*34*	*11.2*
Obesity	NA*	NA*	3	6	3	4.3	1	0.8	*7*	*2.3*
Under nutrition	NA*	NA*	2	4	0	NA	0	NA	*2*	*0.7*
HIV + status	NA*	NA*	13	25.5	4	5.6	9	6.9	*26*	*7.9*
Extrinsic factors										
Central venous catheter	20	37	13	25.5	2	2.8	0	NA	*35*	*11.4*
Peripheral venous catheter	51	94.4	50	98.0	68	95.8	123	93.9	*292*	*95.1*
Urinary catheter	1	1.9	15	29.4	20	28.2	11	8.4	*47*	*15.3*
Nasogastric tube	24	44.4	21	41.2	0	NA	9	6.9	*54*	*17.6*
Intubation	1	1.9	0	NA	0	NA	0	NA	*1*	*0.3*
Surgery	0	NA	4	7.8	48	67.6	79	60.3	*131*	*42.7*
Bedsores	0	NA	12	24.0	0	NA	2	1.5	*14*	*4.6*

*NA* not applicable (impossible to estimate the relative frequency when the absolute one is zero), NA* the variable was not assessed in neonates

Italic values indicate the total value (in all the services).

Fifty-four newborns (17.6%) were recruited from the neonatal unit. All of them were between 0 and 1 month old, with an average age of birth of 38 ± 2.07 weeks and an average weight of birth of 3106 ± 74.7 g. They were 11 (20.37%) premature births, but with 10 (16.52%) low birth weights.

### Incidence of nosocomial infection

Fifty-nine (59) NI were reported from all the 307 participants, hospitalized for a total of 4071 days, giving a cumulative incidence of 19.21% and the incidence rate of 14.49 NI/1000 days. Table 4 presents the distribution by services of the number of NI, the incidence rate, the specific mortality rate of NI as well as time of onset of NI. Intensive care unit was the mostly affected service with the highest specific mortality rate. The mean time of onset of NI was 11 days (SD = 11 days; median = 9 days) and this period was lower in neonatology, 6 days (SD = 2 days, median = 5 days) compared to other services (p = 0.003). Neonates were mostly affected by septicaemia 6 (66.7%) and clinical infections of the catheter 2 (22.2%).

**Table 4:** Description of nosocomial infection in the services

	Number of patients included	Number of cases	Incidence rate (TI)NI/1000 days	Cumulative incidence (IC)	Time of onset (days)^a^	Specific mortality rate (%)
ICU	51	29	23.31	56.86	11	57.1
Surgery	131	14	7.84	10.68	17	14.3
Gynecology	71	6	11.39	1.14	9	0.0
Neonatology	54	9	17.44	1.74	6	0.0
Total	307	59	14.49	19.21	11	28.0

^a^Days post admission to the onset of nosocomial infection

### Types of nosocomial infections

Six types of NI were identified: they included in crescent order of frequency: 1 (1.69%) pneumonia, 8 (13.56%) surgical site infections, 9 (15.25%) urinary tract infections, 12 (20.34%) septicaemia, 12 (20.34%) infections of the skin and soft tissues, and 17 (28.81%) clinical infections of the catheter.

### Types of bacteria isolated

**Fig. 1:**
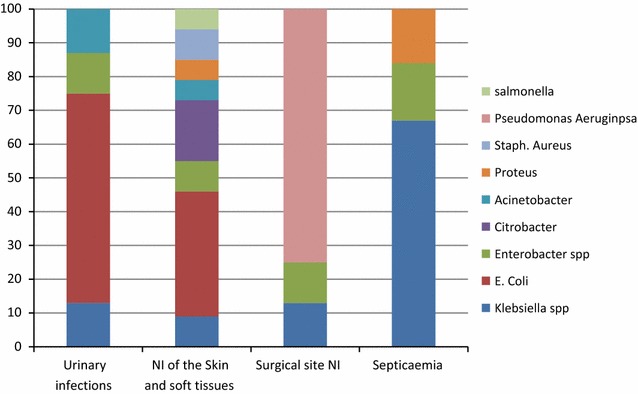
Frequencies of bacteria involved in each type of nosocomial infection

For the 59 infections identified, 42 (71.2%) cultures were performed, and 22 (52.38%) of them positive with 29 micro-organisms isolated. These included the 9 bacteria, namely *Klebsiella* spp., *Escherichia coli*, *Enterobacter* spp., *Citrobacter* spp., *Acinetobacter* spp., *Staphylococcus aureus, Proteus vulgaris*, *Salmonella arizonae* and *Pseudomonas* spp., as shown in Table 5. *Klebsiella* spp., particularly *Klebsiella pneumoniae* was found in all services and in all types of NI recorded during the surveillance. As shown in Fig. 1, *E. coli* was the most frequently isolated bacterium in urinary tract infections and NI of the skin and soft tissues. *Klebsiella* spp*. and Pseudomonas aeruginosa* were frequently isolated in nosocomial septicaemia and NI of the surgical site (Fig. 1).

**Table 5:** Resistance data concerning bacteria

Bacteria	NB of isolate	Antibiotics number (proportion) of isolates resistant											
		Amoxicillin	Chloramphenicol	Gentamycin	Amikacin	Ceftriaxone	Cefotaxim	Fosfomycin	Imipenem	Colistin	Quinolones	Erythromycin	Cotrimoxazol
*E. coli*	7	6 (85.7)	2 (28.6)	0	1 (14.3)	2 (28.6)	4 (57.1)	1 (14.3)	0	2 (28.6)	5 (74.4)	0	4 (57.1)
*Klebsiella* spp.	8	6 (75)	2 (25)	5 (62.5)	0	5 (62.5)	5 (62.5)	1 (12.5)	0	2 (25)	8 (100)	0	5 (62.5)
*Enterobacter* spp.	4	2 (50)	2 (50)	2 (50)	1 (25)	1 (25)	1 (25)	2 (50)	1 (25)	3 (75)	2 (50)	0	2 (50)
*Citrobacter* spp.	4	2 (50)	1 (25)	1 (25)	1 (25)	1 (25)	1 (25)	0	0	1 (25)	1 (25)	0	1 (25)
*Acinetobacter* spp.	2	2 (100)	2 (100)	0	0	2 (100)	0	2 (100)	0	0	2 (100)	0	0
*Proteus* spp.	2	1 (50)	2 (100)	1 (50)	0	1 (50)	2 (100)	0	0	1 (50)	1 (50)	0	0
*Staph. aureus*	1	1 (100)	0	0	0	0	0	0	0	0	1 (100)	1 (100)	1 (100)
*Pseudomonas*	1	1 (100)	0	0	0	0	0	0	0	0	1 (100)	0	1 (100)
*Salmonella*	1	0	0	1 (100)	0	0	1 (100)	0	0	1 (100)	0	0	0
Total	29	21 (72.4)	11 (37.9)	10 (34.5)	3 (10.3)	12 (41.4)	14 (48.3)	6 (20.7)	1 (3.5)	10 (34.5)	21 (72.4)	1 (3.5)	14 (48.3)

(0): none of the isolates showed resistance to this antibiotic

## Discussion

The cumulative incidence (CI) of NI was 19.21% (16.9–21.5). Septicaemia (20%), infection of the skin and soft tissues (20%) and urinary tract infections (15%) were the most frequent type of NI. All bacteria isolated were multi resistant, *Klebsiella* spp. was the most frequently isolated bacterium (27%).

The observed overall CI of NI in this study was in the same range as that of a study conducted in 2010 in the same hospital [9]. From studies conducted in other setting it varies from 6 to 25% [5–7, 12, 13]. Reasons of this variation have not yet been assessed but it may be explained by, the difference in NI frequency in targeted health facilities, methodological differences between studies, including the category of the hospital where the study is conducted, case definition used and sample fluctuation. Our results suggest that the risk of acquiring and NI is high in this particular hospital and that it seems to be higher in intensive care units where NI cumulative incidence was higher (56.8%, P < 0.00001) compared to others. Increased NI incidence rate has been explained from other studies by the absence of resources and guidelines for better control, of training supervision and consequently low level of awareness of health workers, inadequate behaviour [14]. In other settings, providing guidelines, supplies, water and soap, training and supervising health personnel has been documented to contribute in reducing NI incidence rate [10, 15, 16].

The skin and soft tissues were sites with the highest frequency of NI. This does not concord with previous studies in which the surgical site and nosocomial pneumonia had the higher frequencies ranging from 25–35% and 20–45% respectively [8, 17, 18]. This strong representation of skin and soft tissues infections was related to bedsores and suggests a breach of inpatients hygiene in hospitals and aseptic conditions during care. This can be supported by the very low compliance rate to hand wash before care that was retrieved in this hospital [14]. Indeed, it has been documented that continuous staff training on good hygiene practices during care can significantly reduce nosocomial infection rates [10, 19].

Nine bacteria were surveyed, and the majority of these organisms have been documented in the literature but their frequencies differ among studies [20, 21, 23]. *Klebsiella* spp. was the most common germ, followed by *E. coli* and *Enterobacter* spp. Similar results were found in a study conducted in USA and Canada were Klebsiella ranked among the top ten pathogens causing NI and was the third most prevalent pathogen isolated in NI in Latin America [21, 22]. The fact that *Klebsiella* was found in all targeted services shows that this bacteria is in circulation in this Health facility. This can be explained by a possible outbreak of *Klebsiella* in this hospital that is probably spreading from service to service through moving of health workers and visitors. Indeed it has been documented that daily chlorhexidine baths of patients, enhanced environmental cleaning, isolation and contact precautions, training of health workers can help in hospital control of *Klebsiella* [23].

The study showed a predominance of Gram Negative organisms as infecting agents for nosocomial infections in this Health facility. This could be explained, for water derived organisms, by the exposure to unchlorinated tap water used for bathing cleaning of the patients and of bed items. This can be supported by fact that absence of running water and soap has been documented in most services of the same health facilities during a cross sectional study conducted in September 2014 [14]. Also, for the other Gram Negatives that likely have a fecal origin like *E. coli* and *Enterobacter*, lack of patient’s personal hygiene due to lack of access to soap and water can be held the culprit. They can also be endogenous Gram Negative bacteria spread via environmental contamination, especially hands of caregivers. A study conducted in 2014 in this same health facility showed a low compliance rate of health personnel to hand hygiene before and after care [14, 24]. Genetic analysis using PFGE could help determine if these Gram negatives are clonal strains being spread; as PFGE has provided important insights into the epidemiology of many pathogens. Important progress should be made in terms of information and sensitization of health workers in this facility and in other facility of the country, on their role and importance in the potential transmission of NI and the associated morbidity and mortality.

All bacteria found were multi-resistant. This multiresistance can be explained by natural resistance of these bacteria or induced by prescription behaviours in the hospital since for patients lacking resources to pay for their laboratory test, antibiotics prescription is usually only based on presumptive (clinical) diagnostics [25]. A similar increase found by other authors was attributed to epidemic spread of resistance plasmids [17]. These results underline the need to integrate bacteria antibiotics sensitivity in all NI surveillance system. Emphasis must be placed on the rational and judicious use of all antimicrobial agents, as well as having a local antibiogram available for physicians to use systematically when making empiric antibiotic selection.

There were some limitations to this study. NI case definition for all patients was clinical and this limited the detection of asymptomatic cases, the investigation of etiology and risk factors of all cases. Due to insufficient resources, the duration and coverage of the present study was limited if there was a need to infer our results to all patients hospitalized in the targeted health facility in a year. As far as antibiotic resistance is concerned, analysis have been carried out on a relatively small number of bacterial isolates.

## Conclusion

The cumulative incidence of nosocomial infections was 19.21% (16.9–21.5) in this study, with and incidence rate of 14.49 NI/1000 days. This is higher compared to other studies. The incidence varied by type of NI, patient history, duration of hospitalization, inpatient department, and bacteria involved. Strategies need to be identified for a sustainable and continuous monitoring of NI in all health facilities of Cameroon. In addition, further studies should identify NI determinants and interventions for efficient and better control. In the same line a study should be conducted to identify and eliminate the sources of *Klebsiella* spp. in this hospital. The hospital policy and development plan should be revised to provide resources, training and guideline to insure that NI surveillance is continuous and that its results are used to improve the NI control.

## Abbreviations

NI: nosocomial infection
YUTH: Yaoundé University Teaching Hospital
HAIs: healthcare associated infection
SD: standard deviation
RR: relative risk
HIV: human immune deficiency virus
ICU: intensive care unit
CI: cumulative incidence
ASA: American Society of Anesthesiology
Hb: hemoglobin
